# Percolation in protein sequence space

**DOI:** 10.1371/journal.pone.0189646

**Published:** 2017-12-20

**Authors:** Patrick C. F. Buchholz, Silvia Fademrecht, Jürgen Pleiss

**Affiliations:** Institute of Biochemistry and Technical Biochemistry, University of Stuttgart, Stuttgart, Germany; Birla Institute of Technology and Science, INDIA

## Abstract

The currently known protein sequences are not distributed equally in sequence space, but cluster into families. Analyzing the cluster size distribution gives a glimpse of the large and unknown extant protein sequence space, which has been explored during evolution. For six protein superfamilies with different fold and function, the cluster size distributions followed a power law with slopes between 2.4 and 3.3, which represent upper limits to the cluster distribution of extant sequences. The power law distribution of cluster sizes is in accordance with percolation theory and strongly supports connectedness of extant sequence space. Percolation of extant sequence space has three major consequences: (1) It transforms our view of sequence space as a highly connected network where each sequence has multiple neighbors, and each pair of sequences is connected by many different paths. A high degree of connectedness is a necessary condition of efficient evolution, because it overcomes the possible blockage by sign epistasis and reciprocal sign epistasis. (2) The Fisher exponent is an indicator of connectedness and saturation of sequence space of each protein superfamily. (3) All clusters are expected to be connected by extant sequences that become apparent as a higher portion of extant sequence space becomes known. Being linked to biochemically distinct homologous families, bridging sequences are promising enzyme candidates for applications in biotechnology because they are expected to have substrate ambiguity or catalytic promiscuity.

## Introduction

Despite the rapidly growing amount of DNA data due to advances in DNA sequencing techniques, only a tiny fraction of all protein sequences existing in the biosphere has been sequenced, yet. While we currently know the sequences of almost 10^8^ proteins [1], the number of extant sequences was estimated to be 10^34^, and up to 10^43^ different protein sequences might have been explored during 4 Gyr of evolution [2]. Though this number seems to be large, it is infinitesimally small as compared to the theoretical sequence space (10^400^ possible sequences for a medium-sized protein), and it would be highly improbable to find functional proteins by random search [3]. Therefore, the Darwinian model of protein evolution based on mutation of the genotype and subsequent natural selection of the phenotype excludes the possibility of extant sequences being scattered in the theoretical sequence space, but they are expected to form a connected network, where functional sequences and mutations form the nodes and edges, respectively [4]. In his fundamental article about the structure of sequence space, J. Maynard Smith asked the questions whether indeed all existing proteins are part of a single network with a single starting point, what fraction of the functional sequence space has been explored yet, and how large is the space of functional, but never-born proteins [5]. Although the sequence space of functional proteins is unknown, we can reliably measure distances between sequences by global or local alignment methods [6]. The currently known protein sequences are not equally distributed, but cluster into homologous families [7]. However, due to the sparsity of the known sequence space, in most clusters even neighboring nodes differ by multiple mutations. As an exception, the TEM β-lactamase family has a very high microdiversity, and the variants form a dense single network with nodes connected by single mutations [8].

The apparent sparsity of the known sequence space is a consequence of our limited knowledge of the extant sequences in the biosphere. Therefore, we expect that as we know more sequences, all nodes will gradually form a connected network. As an alternative explanation of sparsity, the observed separation between clusters is the consequence of ancestor sequences having become extinct during evolution [9].

In this work, the known sequence space was explored by applying percolation theory to learn about the extant sequence space. Percolation theory describes the cluster distribution on a randomly populated lattice, with a parameter p describing the occupancy of the lattice sites [10]. For increasing values of p, the characteristic cluster size s_ξ_ and the fraction P of sites belonging to the largest cluster increases. As p approaches the percolation threshold p_c_, an infinite cluster appears for the first time on an infinite lattice, while on a finite-sized lattice the largest cluster percolates between the lattice boundaries. The core of percolation theory is a set of scaling relations that depend on |p_c_-p|, such as s_ξ_ ∼ |p_c_-p|^-1/σ^ and P ∼ (p-p_c_)^β^ with critical exponents σ and β which depend on the geometry of the lattice. Most importantly, percolation theory predicts that the cluster size distribution N(s) (the number N of clusters with size s) decreases for s << s_ξ_ as N(s) ∼ s^-τ^ and decays exponentially for s >> s_ξ_. Near to percolation (p→p_c_), s_ξ_ becomes infinite. Thus, for s spanning many orders of magnitude log N(s) depends linearly on log s, with the Fisher exponent τ describing the ratio of small to large clusters.

Thus, investigating the cluster size distribution N(s) of homologous protein families provides insights into the structure of the known sequence space and gives a glimpse of the extant sequence space, despite our limited knowledge.

## Materials and methods

### Clustering

The in-house databases on α/β hydrolases (abH, 395000 sequences)[11], cytochrome P450 monooxygenases (CYP, 53000 sequences)[12], thiamine diphosphate-dependent decarboxylases (DC, 39000 sequences)[13], and β-hydroxyacid dehydrogenases / imine reductases (bHAD, 31000 sequences)[14] were updated by searching the NCBI non-redundant protein database (GenBank [15]) by *BLAST* [16]. For each homologous family, representative sequences were selected as seed sequences. Family databases for short-chain dehydrogenases/reductases (SDR, 141000 sequences) and ω-transaminases (oTA, 121000 sequences) were established based on seed sequences derived from literature [17],[18].

For each protein database, sequence identities of high-scoring sequence pairs were calculated by the USEARCH software suite (version 9.2)[19]. Sequence pairs with a distinct sequence identity cutoff were clustered by the Python module graph-tool (*https://graph-tool.skewed.de/*) (version 2.17).

### Cluster size distribution

For the six protein superfamilies, the cluster size distribution N(s) was analyzed for cluster sizes s = 1, 2, 3,…, 1000. Because for large cluster sizes data becomes increasingly sparse, a histogram distribution was generated by counting the number of clusters N_i,j_ = Σ ^j^_s = i,_ N(s) with cluster sizes between i and j.

The observed cluster size distributions were compared to three model distributions: a Gaussian distribution N(s) ~ exp(-½·(s-μ)^2^/σ^2^), an exponential distribution N(s) ~ exp(-b·s) and a power law distribution N(s) ~ s^-τ^ with the Fisher exponent τ characterizing the model distribution (**S1 Fig**). Excel sheets for the calculation of the distributions are provided as supporting information (S1 File). The log-log plots of the three model distributions differ considerably: log N(s) of the Gaussian distribution increases gradually with log s and decays rapidly for s>μ, while for the exponential distribution is decays rapidly for all s>0. In contrast, for the power law distribution log N(s) depends linearly on log s with a slope of –τ.

For each model distribution, the respective histogram distribution was calculated. Qualitatively, the histogram distributions were similar to the model distributions. For power law distributions with τ>1, the corresponding histogram distribution could also be approximated by a straight line with a slope of -τ_h_. However, the two slopes -τ and -τ_h_ deviated.

For each histogram distribution of the six protein families, the slope -τ_h_ was determined by fitting the initial linear decay (N_1,10,_ N_11,100_, and N_101,1000)_ by linear regression, and the Fisher exponent of the respective cluster size distribution was derived from τ_h_ by varying τ of the model distribution to fit the observed τ_h_.

## Results

### Sequence space

The known protein sequence space is rapidly increasing, but it represents only a tiny fraction of the extant sequence space, that has been explored during evolution. In turn, the extant sequence space represents a fraction p of the much bigger sequence space coding for functional proteins. Although both the extant and the functional sequence space and therefore also p are unknown, the scaling properties of the cluster size distribution can be used as an indicator of p: if the cluster size distribution in the extant sequence space follows a power law over many orders of magnitude, p is close to a critical percolation threshold p_c_.

The scaling properties of the extant sequence space are investigated by analyzing the scaling properties of the much smaller space of known sequences. Because a typical protein superfamily consists of 10^4^−10^5^ protein sequences, the cluster size range is limited to 2–3 orders of magnitude. The sparsity of the known sequence space has three major consequences: (1) Because of the poor statistics of the cluster size distribution N(s) between s = 1 and 1000, the number of clusters with a size between 1 and 10 (N_1,10_), 11 and 100 (N_11,100_), and 101 and 1000 (N_101,1000_) are analyzed, and the corresponding cluster size distribution is derived from this histogram distribution. (2) Except for very few families, e.g. TEM β-lactamases, it is rare that two members of a protein superfamily differ by only one amino acid. Therefore, neighbor relationships are established by global sequence identity as a cutoff criterion. Using a 90% cutoff criterion, two proteins of 400 amino acids are considered to be neighbors if they differ in less than 40 positions. As a consequence, the structure of the resulting network and the Fisher exponent τ depend on the cutoff criterion for the neighborhood relationship. (3) The Fisher exponent τ depends on the number of known sequences. As the number of known sequences increases, the protein families become more densely populated, and the number of large clusters is expected to increase. As a consequence, the Fisher exponent τ decreases. Therefore, the observed Fisher exponent τ as evaluated from the known protein superfamilies represents an upper limit to the Fisher exponent of the extant sequence space.

**Fig 1:**
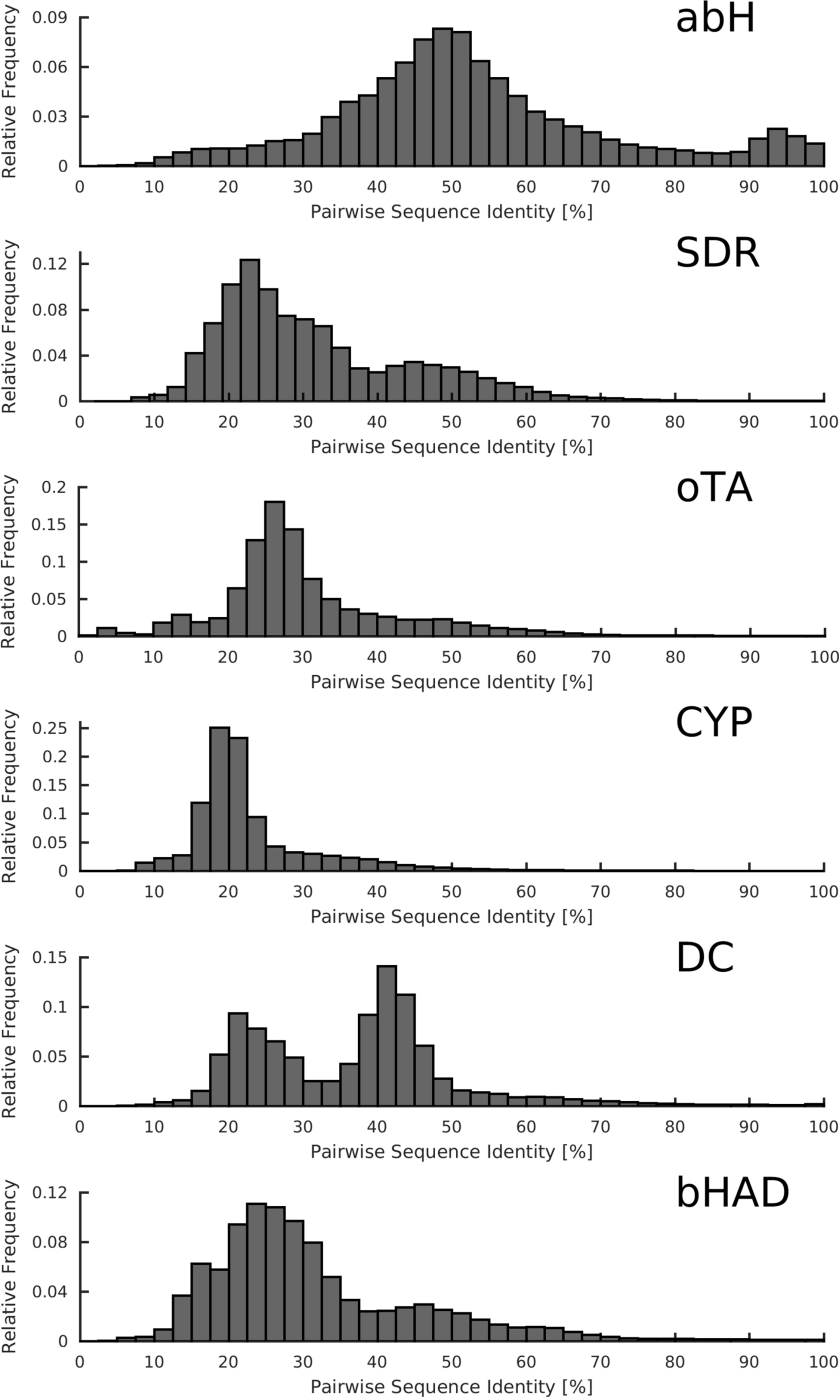
Distributions of pairwise global sequence identity. Distributions of pairwise global sequence identity for the protein families of α/β-hydrolases (abH), short-chain dehydrogenases/reductases (SDR), ω-transaminases (oTA), cytochrome P450 monooxygenases (CYP), thiamine diphosphate-dependent decarboxylases (DC) and β-hydroxyacid dehydrogenases/imine reductases (bHAD).

The structure of the known sequence space was analyzed for six large protein superfamilies with high diversity in sequence and function: α/β hydrolases (abH, 395,000 sequences) [11], short-chain dehydrogenases/reductases (SDR, 141,000 sequences), ω-transaminases (oTA, 121,000 sequences), cytochrome P450 monooxygenases (CYP, 53,000 sequences) [12], thiamine diphosphate-dependent decarboxylases (DC, 39,000 sequences) [13], and β-hydroxyacid dehydrogenases / imine reductases (bHAD, 31,000 sequences) [14] (Table 1**)**. The six protein superfamilies differ in their fold and their number of family members, which is reflected in the distributions of pairwise sequence identity (**Fig 1)**. In the abH superfamily, the majority of sequences had pairwise sequence identity of 40–60%, while almost all CYPs had a pairwise sequence identity of 15–25%. SDRs, DCs and bHADs showed a bimodal distribution with maxima at 20–30 and 40–50%.

**Table 1:** Protein superfamily size and the Fisher exponent extrapolated to 100% sequence identity (τ_100_) of the six protein families.

Abbreviation	Enzyme superfamily	Superfamily size	τ_100_
abH	α/β hydrolases	395000	2.6
SDR	short-chain dehydrogenases/reductases	141000	2.4
oTA	ω-transaminases	121000	2.3
CYP	cytochrome P450 monooxygenases	53000	3.3
DC	thiamine diphosphate-dependent decarboxylases	39000	2.8
bHAD	β-hydroxyacid dehydrogenases/imine reductases	31000	2.5

### Cluster size distribution

**Fig 2:**
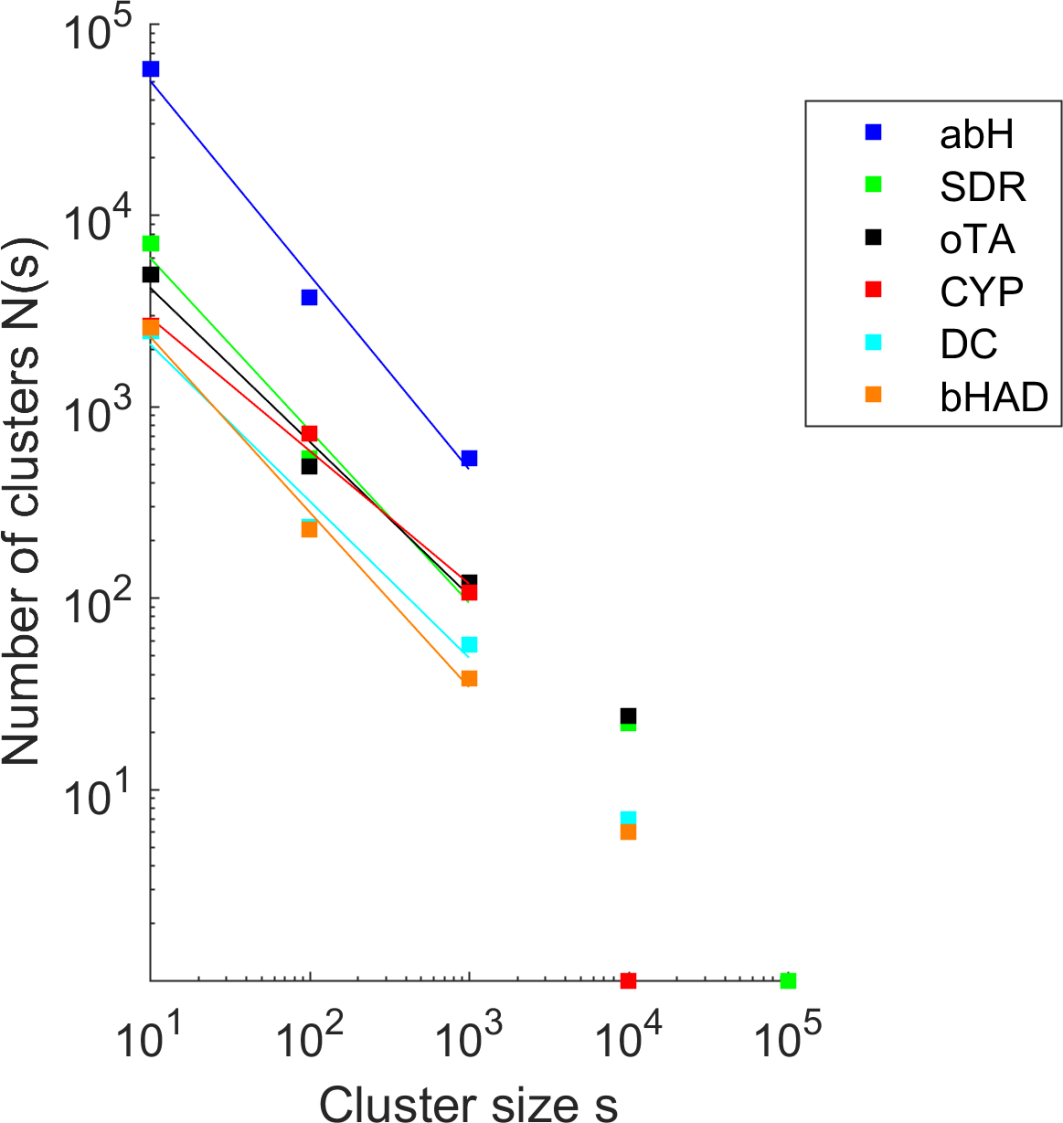
Cluster size distributions. Cluster size distribution of α/β hydrolases (abH), short-chain dehydrogenases/reductases (SDR), ω-transaminases (oTA), cytochrome P450 monooxygenases (CYP), thiamine diphosphate-dependent decarboxylases (DC), and β-hydroxyacid dehydrogenases/imine reductases (bHAD) follow a power law distribution: N(s) ~s^-τ^ (N(s), number of clusters of size s; τ, Fisher exponent). Cluster criterion: 60% global sequence identity.

For each of the six protein superfamilies, the sequences were clustered by a cutoff criterion of 60% global sequence identity which is often applied for defining homologous families. The number N of clusters with size s was analyzed in a histogram with logarithmic bins for s between 1 and 10, 11 and 100, 101 and 1,000, 1,001 and 10,000, and 10,001 and 100,000 to improve statistical sampling (**Fig 2**). Intuitively, we had expected a Gaussian normal distribution, assuming a random distribution of cluster sizes. However, in contrast to intuition, the distribution of cluster sizes followed a power law N(s) ~ s^-τh^, indicated by a linear dependency of log s and log N(s) for the six protein superfamilies (abH, SDR, oTA, CYP, DC, bHAD). The Fisher exponent τ_h_ of a histogram describes the ratio between small and large clusters and is derived from linear regression in the log-log plot of the histogram [20]. From the Fisher exponent τ_h_ of the histogram, the Fisher exponent τ of the underlying cluster size distribution was calculated by fitting the observed τ_h_ of the histogram to a model distribution of cluster sizes following a power law distribution. Though the protein families differ in size, structure, and function, for four of the five (SDR, oTA, DC, bHAD) the Fisher exponent τ varied only slightly (1.8–1.9). The smallest Fisher exponent was derived for the CYP superfamily (τ = 1.6). For the largest superfamily (abH), the Fisher exponent was 2.0. These values are in agreement with the Fisher exponent of τ≈2 determined for the protein family size distribution of the Gene3D database [21] or the TRIBES resource [22], while the distribution of protein folds showed a slightly larger exponent of 2.5 [23].

### Dependency of τ on the cluster criterion

**Fig 3:**
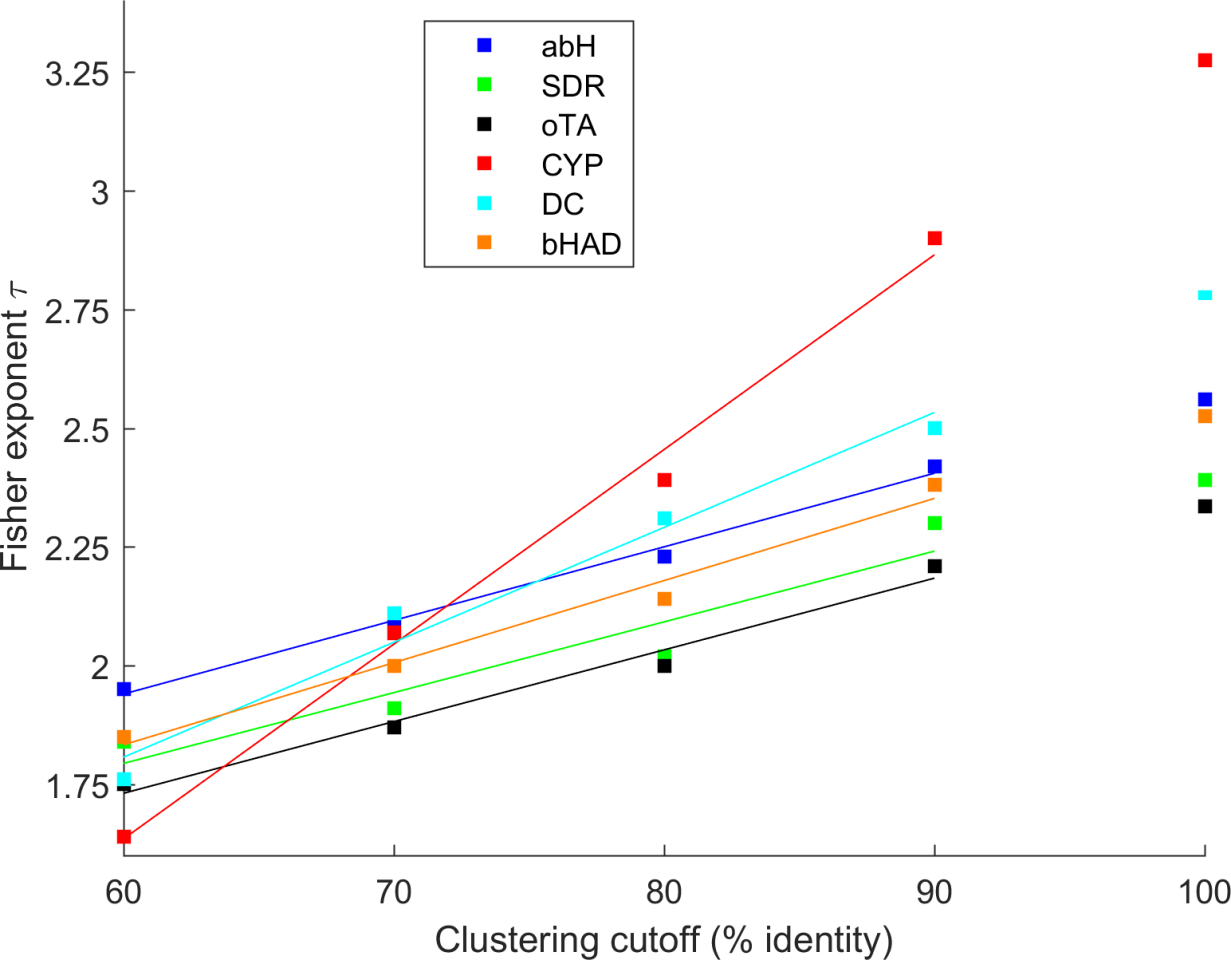
Fisher exponents. Fisher exponent τ of the size distribution of homologous families for clustering cutoffs between 60 and 90% with extrapolated Fisher exponent τ_100_ determined by linear regression (abbreviations according to Fig 2).

While the Fisher exponent τ was almost independent of the protein family and its size, its absolute value depended on the cutoff criterion used for clustering. Upon clustering of the six families with six cutoffs between 60 and 90%, the cluster size distributions followed a power law for all cutoffs (**S2 Fig**). With increasing clustering cutoff, the relative number of small clusters increases, while the number of large clusters decreases. Consequently, the Fisher exponent τ increased almost linearly with increasing cutoff (**Fig 3**) from τ_60_ = 1.6–2.0 at 60% cutoff, to τ_90_ = 2.2–2.9 at 90% cutoff. The Fisher exponent τ was extrapolated to a cutoff of 100%, representing a network of nodes separated by only one mutation (τ_100_). For the six protein families, the extrapolated τ_100_values varied between 2.4 and 3.3 (2.6, 2.4, 2.3, 3.3, 2.8 and 2.5 for abH, SDR, oTA, CYP, DC, and bHAD, respectively).

### Dependency of τ on the number of sequences

Of the 395,000 abH sequences, 50, 25, or 12.5% were randomly selected and clustered, and the cluster size distribution was determined for four distinct cutoff values (**S3 Fig**). With a decreasing number of sequences, the relative number of small clusters increased, while the number of large clusters decreased. Consequently, the Fisher exponent τ increased with decreasing number of sequences: at 60% cutoff from 2.0 for the complete database to 2.0, 2.2, and 2.3 at 50, 25, and 12.5% randomly selected abH sequences, respectively. A similar trend was observed for the other cutoff values: τ_70_ = 2.1–2.5, τ_80_ = 2.2–2.9, τ_90_ = 2.4–3.2. Therefore, it is expected that the Fisher exponent τ of the cluster distribution of the known sequences decreases as more extant sequences will be sequenced in the future, and the extrapolated τ_100_ values for the six families (between 2.4 and 3.3) represent upper limits to the cluster size distribution of the extant sequence network. Because percolation theory predicts values of τ between 2.055 for percolation in a 2-dimensional lattice and 2.5 in a lattice with more than 5 dimensions [24], the upper limits of 2.4–3.3 are in agreement with percolation in the extant sequence space.

Thus, the observation of a power law cluster size distribution results from the connectedness of extant sequence space which is as a consequence of Darwinian evolution. Interestingly, a model that describes protein structural evolution on a three dimensional lattice also results in a power law cluster size distribution with an exponent of 2.3 [25]. It is a tempting observation that the two foundations of protein evolution, the connectedness of extant sequence space and the formation of a stable fold, both result in a power law cluster size distribution with a similar exponent. This observation relates to the fundamental property of protein folds: the stability of a fold is closely related to its evolvability. The more stable a fold is, the more sequences can adopt it, thus forming larger and better connected sequence networks.

## Discussion

### Connectedness and saturation of sequence space

The cluster size distribution of the known sequence space of six protein superfamilies followed a power law, with the extrapolated Fisher exponent τ_100_ being an upper limit to the Fisher exponent of the extant sequence space. The observation of few clusters containing many sequences might relate with the assumption that more stable protein folds are more evolvable, thus forming larger and higher connected clusters of mutations. The extrapolated Fisher exponent is independent of characteristic properties of the protein families such as family size (Table 1). Because the Fisher exponent measures the ratio of small to large clusters, it can be interpreted as an indicator of the global connectedness of the known sequences of a protein family. The protein families oTA, SDR, bHAD and abH (τ_100_ = 2.3, 2.4, 2.5 and 2.6, respectively) had a smaller τ_100_ and thus a higher ratio of large to small clusters than the protein families DC, or CYP (τ_100_ = 2.8 and 3.3, respectively). A high ratio of large to small clusters indicates a high connectedness. There are at least three possible reasons for a high connectedness of a protein family: (1) The protein family is well explored; thus, a high fraction of its extant sequence space is already known. (2) The protein family has a high microdiversity. (3) The protein family covers only a small region in sequence space, thus overall variability is low.

Our observation that the connectedness gradually increased as more sequences become known is supported by the concept of gradual saturation of sequence space. This concept describes the observation that the number of newly sequenced genes that form separate clusters plotted over time decreases to zero[26]. Rather than expanding, the sequence space of protein families is gradually becoming denser and more connected. As τ_100_ measures the connectedness of the protein family, it also measures the current level of saturation, with the protein families SDR and CYP having the highest and lowest saturation, respectively.

### Bridges between homologous families

The six protein families showed a similar linear dependency of τ on the clustering cutoff. Thus, for high cutoff values many small clusters were observed, which gradually combine into larger clusters as the clustering cutoff was decreased, and bridges between clusters gradually appeared (**S4 and S5 Figs**). These bridges were formed by sequences that had been part of one cluster and then became part of a second cluster, or were recruited from previously isolated sequences, as the clustering cutoff was decreased. These bridging sequences are interesting, as they belong to both clusters. If global sequence similarity relates to biochemical function, a cluster is characterized by a similar biochemical function that differs between the clusters. The bridging sequences, having similarities to two or even more clusters, are therefore promising candidates with substrate ambiguity [27] or even catalytic promiscuity [28].

### Protein evolution

By analyzing the known sequence space, we predict that extant proteins form a percolating, highly connected network where each sequence has multiple neighbors, and each pair of sequences is connected by many different paths, as expected from evolution [4]. However, the density in sequence space is not uniform, but follows a power law distribution which indicates that certain folds were more evolvable than others. Percolation allows for the concept of evolution as adaptive walks on a fitness landscape [29], where sequences at the ends of the walks may substantially differ from one another [30]. A high degree of connectedness also overcomes the possible blockage by sign epistasis and reciprocal sign epistasis [31] and thus is a necessary condition of efficient evolution, despite the fact that only an infinitesimally small portion of the theoretical sequence space been explored during the course of life on Earth [2]. In a highly connected sequence network as a model of evolution [32], sequences are found that form bridges between two clusters. Since the number of bridges is much smaller than the number of cluster members, they only gradually appear as the number of sequenced genes increases. Consequently, the observed separation of families is merely a consequence of our limited knowledge of extant sequence space. With increasing sequence data from genomics and metagenomics projects, we expect more and more sequences to occur which form bridges between yet separated families and thus contribute to the connectedness of known sequence space.

These bridging sequences are equivalent to reconstructed ancestral sequences in binary trees [33]. Since they form a link between two branches, ancestral proteins are assumed to be generalists with a broader substrate spectrum or even multiple activities [28]. While the binary tree model of evolution assumes that the ancestor sequences have disappeared from the biosphere, the network model of evolution assumes that bridging sequences still exist. For any two neighboring, biochemically distinct clusters, we expect bridging sequences to exist that contribute to the formation of a continuous network. It will be challenging to analyze how the biochemical properties change as we walk across the bridges. Most probably, bridging sequences are multi-functional or promiscuous enzymes with known or latent activities of both sub-families. In contrast to ancestors, these generalists already exist in the biosphere and are waiting to be found.

## Supporting information

S1 Fig Model distributions. Model distributions displayed as log-log plot: Gaussian distribution N(s) = a·exp(-½ (s-μ)^2^/σ^2^) with a = 10000, μ = 200, σ = 50, exponential distribution N(s) = a exp(-b s) with a = 10000 and b = 0.2, power law distribution N(s) = a·s^-τ^ with a = 10000, -τ = 2.5.
(TIF)

S2 Fig Cluster size distributions. Cluster size distributions for 60, 70, 80, and 90% global sequence identity of the six protein superfamilies from Table 1 (α/β-hydrolases in blue, short-chain dehydrogenases/reductases in green, ω-transaminases in black, cytochrome P450 monooxygenases in red, thiamine diphosphate-dependent decarboxylases in cyan and β-hydroxyacid dehydrogenases/imine reductases in orange).
(TIF)

S3 Fig Cluster size distributions of subsets. Cluster size distributions for 60, 70, 80, and 90% global sequence identity of all abH sequences (filled squares) and randomly selected abH sequences: 50% (open squares), 25% (filled circles) and 12.5% (open circles) of the original dataset.
(TIF)

S4 Fig Sequence identity networks with clustering cutoff at 39% sequence identity. Details of sequence identity networks for two homologous families of short-chain dehydrogenases/reductases (SDR) with clustering cutoff at 39% sequence identity. The network shows bridges connecting the two homologous families (indicated in red hexagons). Visualization in Cytoscape (version 3.2.1) using organic layout.
(PNG)

S5 Fig Sequence identity networks with clustering cutoff at 40% sequence identity. Details of sequence identity networks for two homologous families of short-chain dehydrogenases/reductases (SDR) with clustering cutoff at 40% sequence identity. The bridge sequences from **S4 Fig** are indicated in red hexagons. Visualization in Cytoscape (version 3.2.1) using organic layout.
(PNG)

S1 File Model distributions. Power law, Gauss, and exponential model distributions.
(XLSX)
