# Supplementary figures and images for: Percolation in protein sequence space
Source: PLoS One. 2017 Dec 20;12(12):e0189646. doi: 10.1371/journal.pone.0189646 (PMC5738032; doi:10.1371/journal.pone.0189646)

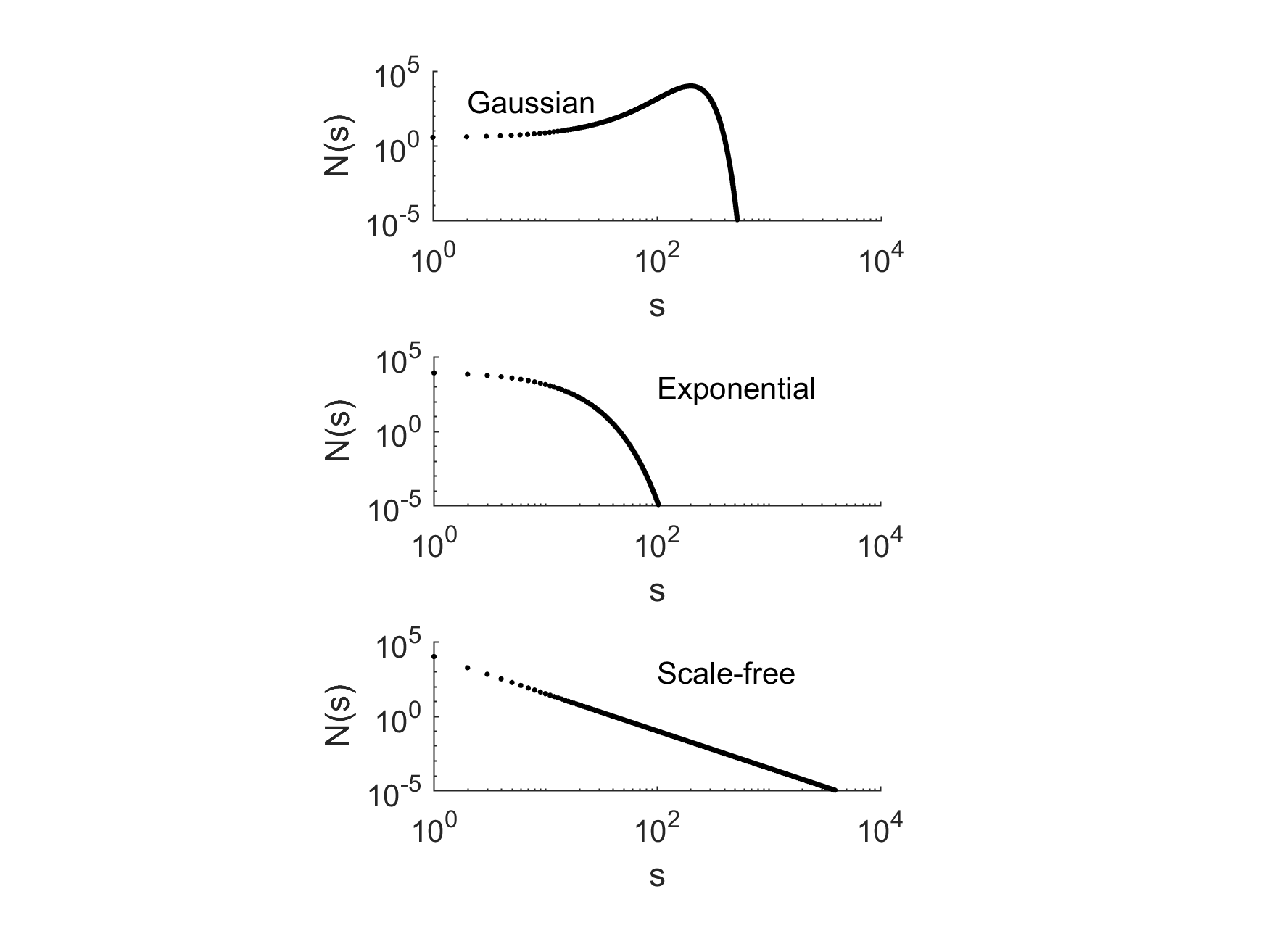

Supplement: S1 Fig — Model distributions displayed as log-log plot: Gaussian distribution N(s) = a·exp(-½ (s-μ)2/σ2) with a = 10000, μ = 200, σ = 50, exponential distribution N(s) = a exp(-b s) with a = 10000 and b = 0.2, power law distribution N(s) = a·s-τ with a = 10000, -τ = 2.5. (TIF) [file pone.0189646.s001.tif]

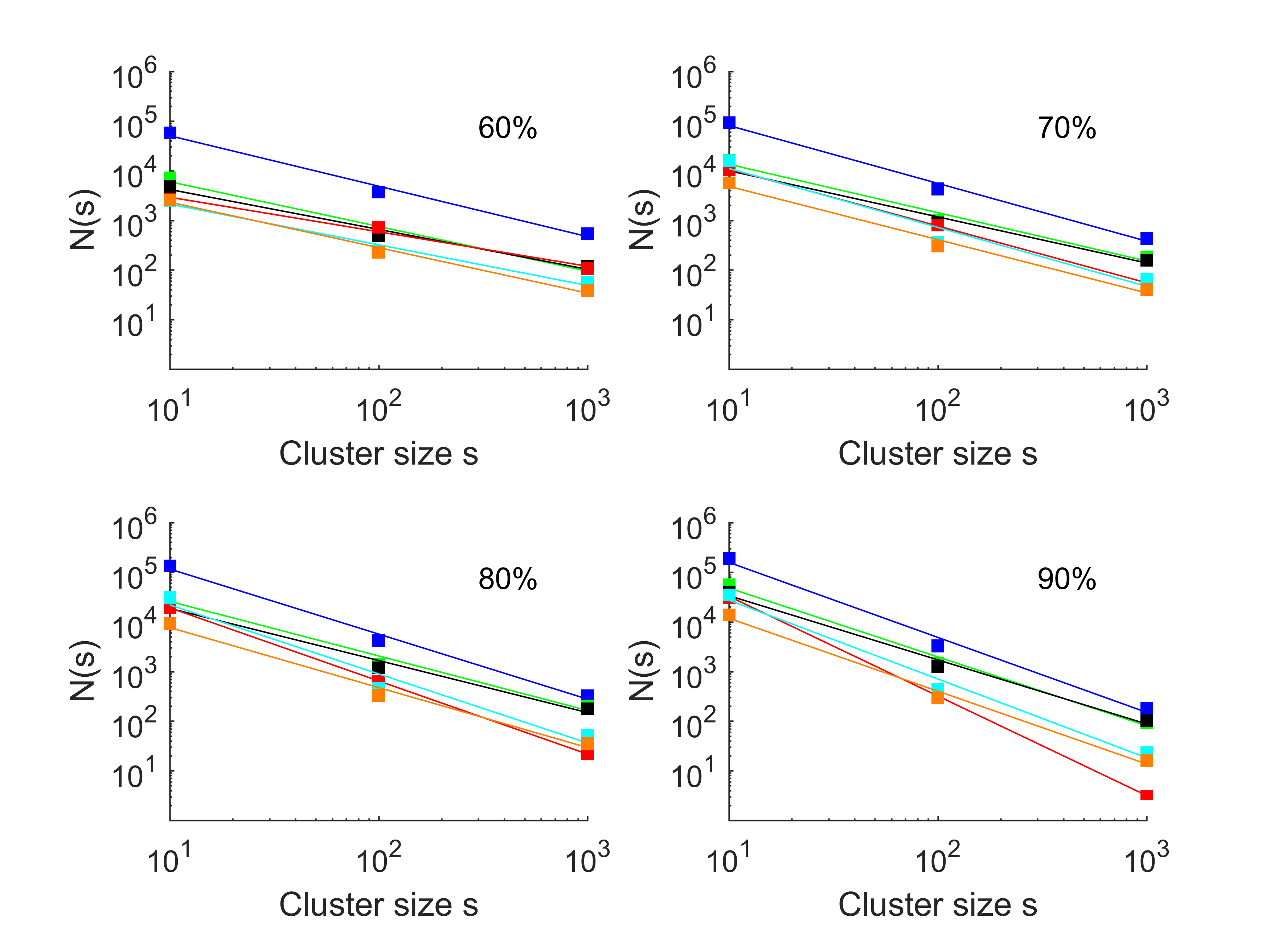

Supplement: S2 Fig — Cluster size distributions for 60, 70, 80, and 90% global sequence identity of the six protein superfamilies from Table 1 (α/β-hydrolases in blue, short-chain dehydrogenases/reductases in green, ω-transaminases in black, cytochrome P450 monooxygenases in red, thiamine diphosphate-dependent decarboxylases in cyan and β-hydroxyacid dehydrogenases/imine reductases in orange). (TIF) [file pone.0189646.s002.tif]

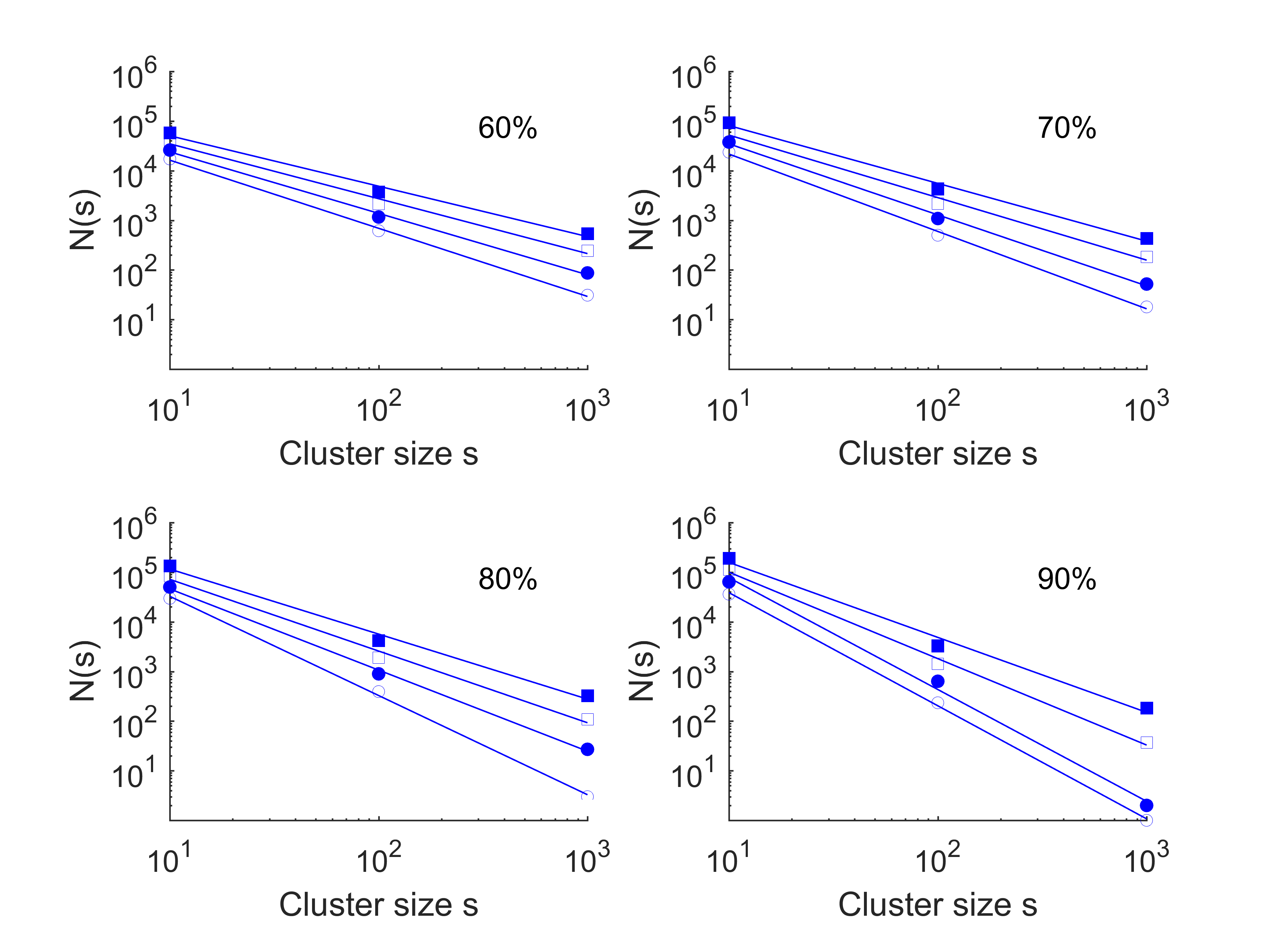

Supplement: S3 Fig — Cluster size distributions for 60, 70, 80, and 90% global sequence identity of all abH sequences (filled squares) and randomly selected abH sequences: 50% (open squares), 25% (filled circles) and 12.5% (open circles) of the original dataset. (TIF) [file pone.0189646.s003.tif]

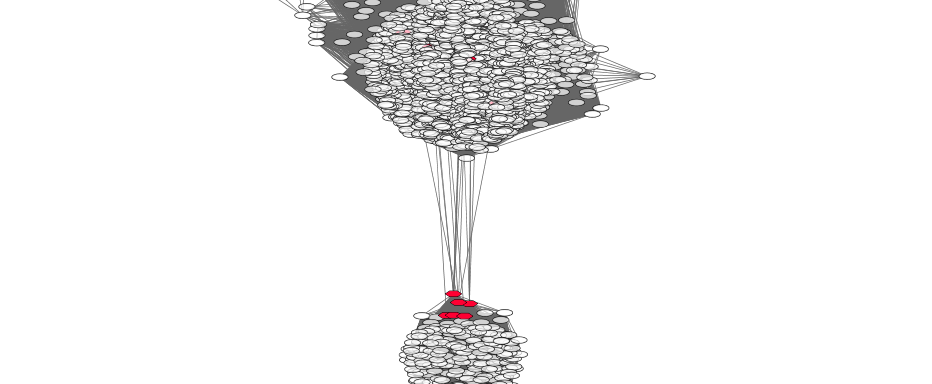

Supplement: S4 Fig — Details of sequence identity networks for two homologous families of short-chain dehydrogenases/reductases (SDR) with clustering cutoff at 39% sequence identity. The network shows bridges connecting the two homologous families (indicated in red hexagons). Visualization in Cytoscape (version 3.2.1) using organic layout. (PNG) [file pone.0189646.s004.png]

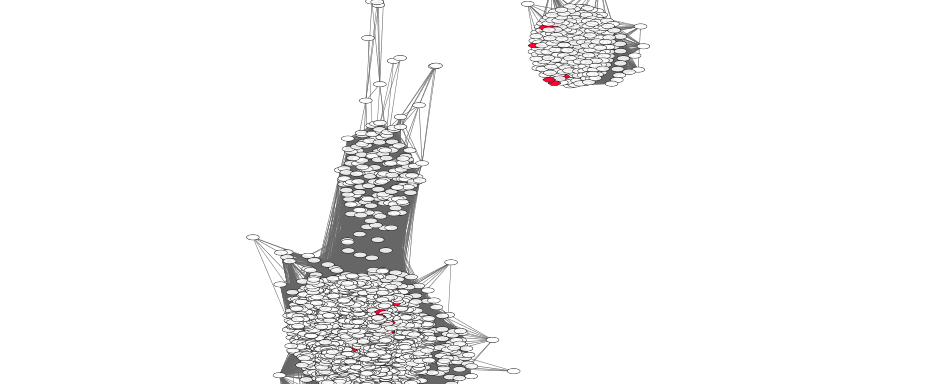

Supplement: S5 Fig — Details of sequence identity networks for two homologous families of short-chain dehydrogenases/reductases (SDR) with clustering cutoff at 40% sequence identity. The bridge sequences from S4 Fig are indicated in red hexagons. Visualization in Cytoscape (version 3.2.1) using organic layout. (PNG) [file pone.0189646.s005.png]
